# Targeting ferroptosis unveils a new era for traditional Chinese medicine: a scientific metrology study

**DOI:** 10.3389/fphar.2024.1366852

**Published:** 2024-02-14

**Authors:** Siyang Cao, Yihao Wei, Yaohang Yue, Yingqi Chen, Shuai Liao, Aikang Li, Peng Liu, Ao Xiong, Hui Zeng

**Affiliations:** ^1^ National and Local Joint Engineering Research Centre of Orthopaedic Biomaterials, Peking University Shenzhen Hospital, Shenzhen, Guangdong, China; ^2^ Shenzhen Key Laboratory of Orthopaedic Diseases and Biomaterials Research, Peking University Shenzhen Hospital, Shenzhen, Guangdong, China; ^3^ Department of Bone and Joint Surgery, Peking University Shenzhen Hospital, Shenzhen, Guangdong, China; ^4^ Shenzhen Second People’s Hospital, The First Affiliated Hospital of Shenzhen University, Shenzhen, Guangdong, China

**Keywords:** global scientific frontiers, ferroptosis, Traditional Chinese Medicine, bibliometrics, visualized analysis

## Abstract

In the past 11 years, there has been a surge in studies exploring the regulatory effect of Traditional Chinese Medicine (TCM) on ferroptosis. However, a significant gap persists in comprehensive scientometric analysis and scientific mapping research, especially in tracking the evolution, primary contributors, and emerging research focal points. This study aims to comprehensively update the advancements in targeting ferroptosis with various TCMs during the previous 11 years. The data, covering the period from 1 January 2012, to 30 November 2023, were retrieved from the Web of Science database. For in-depth scientometric and visualized analyses, a series of advanced analytical instruments were employed. The findings highlight China’s predominant role, accounting for 71.99% of total publications and significantly shaping research in this domain. Noteworthy productivity was observed at various institutions, including Guangzhou University of Chinese Medicine, Chengdu University of Traditional Chinese Medicine, and Zhejiang University. Thomas Efferth emerged as the foremost author within this field, while *Frontiers in Pharmacology* boasted the highest publication count. This study pinpointed hepatocellular carcinoma, chemical and drug-induced liver injury, mitochondrial diseases, acute kidney injury, and liver failure as the most critical disorders addressed in this research realm. The research offers a comprehensive bibliometric evaluation, enhancing our understanding of the present status of TCM therapy in managing ferroptosis-related diseases. Consequently, it aids both seasoned researchers and newcomers by accelerating access to vital information and fostering innovative concept extraction within this specialized field.

## 1 Introduction

Ferroptosis represents a novel form of programmed cell death (PCD), characterized by elevated intracellular iron levels and the accumulation of lipid reactive oxygen species within the cell (Dixon et al., 2012). Over the last 11 years, a global accumulation of evidence has emerged, highlighting the potential crucial role of ferroptosis in various biological processes such as tumor suppression and immunity (Sun et al., 2023). This underscores the significance of ferroptosis in health maintenance, achieved through the regulation of metabolism and redox homeostasis. Recent research has unveiled ferroptosis’s critical involvement in various pathophysiological processes, including ischemic organ injury, stroke, cardiac myopathy, and neurodegenerative diseases. Moreover, ferroptosis has been implicated in various oncogenic pathways, suggesting its potential as an innovative target for cancer therapeutics (Friedmann Angeli et al., 2019; Wang X. et al., 2023). The annual publication of studies on ferroptosis is currently experiencing exponential growth (Stockwell, 2022). By the end of 2023, more than 10,000 papers worldwide had been published on this topic (https://www.ncbi.nlm.nih.gov), emphasizing its significance in maintaining health through the regulation of iron/lipid metabolism and glutathione (GSH)-dependent redox homeostasis (Stockwell, 2022; Wang X. et al., 2023; Sun et al., 2023). Considering the potential therapeutic relevance of ferroptosis regulatory mechanisms, the refinement of ferroptosis-mediated therapeutics holds profound clinical significance for treating a spectrum of diseases (Sun et al., 2023).

Traditional Chinese Medicine (TCM) stands as an extensive reservoir of practical clinical knowledge amassed over millennia, significantly contributing to human health (Pitschmann et al., 2013; Gao et al., 2022). It finds its roots in Chinese philosophy, emphasizing the harmonious equilibrium between humanity and the natural world. TCM encompasses fundamental theories including Yin and Yang, the Five Elements, zang-fu, channels-collaterals, qi, blood, body fluid, diagnostic methods, and the differentiation of symptom-complexes (Hao and Keji, 2003). It distinguishes itself through holistic treatment strategies, emphasizing syndrome differentiation and utilizing natural products guided by taste, Yin-Yang qualities, and active pharmaceutical ingredients (Chan, 1995; Dixon et al., 2012; Cragg and Newman, 2013). With its diverse components, objectives, connections, and pathways, TCM presents distinct clinical advantages in both disease prevention and treatment (Wang et al., 2020; Shi et al., 2023). In 2018, a noteworthy achievement was reached when the World Health Organization, for the inaugural time, incorporated TCM into its influential worldwide medical compendium (Wu et al., 2021). In the realm of oncology, particular active pharmaceutical ingredients in TCM act as ferroptosis inducers, inducing PCD and eliminating malignant cells (Wang et al., 2023b). Conversely, specific components of TCM serve as ferroptosis inhibitors, reducing cytotoxic damage in healthy cells like chondrocytes, neurons, hepatocytes, and cardiomyocytes due to irregularities in iron/GSH/lipid metabolism (Wang et al., 2020; Wu et al., 2021; Wu et al., 2022; Zhao et al., 2022; Cao et al., 2023a). This characteristic is in alignment with the TCM philosophical concept of Yin and Yang, which encompasses unity, opposition, and mutual transformation.

Although several reviews on “ferroptosis-TCM” have been published (Wang et al., 2020; Wu et al., 2021; Gao et al., 2022; Liu et al., 2022; Wu et al., 2022; Zhao et al., 2022; Zhang X. et al., 2023; Wang et al., 2023b; Ding et al., 2023; Shi et al., 2023; Xie et al., 2023), there remains a significant need for comprehensive scientometric analyses and detailed summaries encompassing developmental trends, principal institutions/authors, and central research themes. Therefore, this study aims to comprehensively update the advancements in targeting ferroptosis with various TCMs during the previous 11 years. Aiming to be a significant resource for both established experts and novices, this study offers thorough evaluations, identifies emerging research trends, and informs future research strategies. Adopting a visualization-focused approach significantly enhances research productivity and effectiveness in this domain. The current study, as far as we know, provides the first comprehensive analysis of this topic from a scientometric viewpoint.

## 2 Methods

### 2.1 Methodology for data retrieval

Widely embraced for its standardized, high-quality academic publication data, the Web of Science Core Collection (WoSCC) (https://www.webofscience.com/wos/) is a renowned resource in bibliometric analysis for tracking the evolution of scientific frontiers (Boudry et al., 2018; Zhang XL. et al., 2020; Pei et al., 2022; Ling et al., 2023). It provides bibliometric software with a source of general statistics (Pei et al., 2022), and the accuracy of document type labeling in the WoSCC has been demonstrated to be superior to that of other databases (Yeung, 2019). This study involved a comprehensive online search within the WoSCC, with a focus on original researches and reviews pertaining to “ferroptosis-TCM”. The search covered publications from 1 January 2012, to 30 November 2023, using both Medical Subject Heading terms and free words for data extraction. The search strategy was refined through multiple iterations by three researchers (SYC, AKL, and SL) to fortify its sensitivity and precision. The Supplementary Materials contain an extensive exposition of the search methodology.

### 2.2 Inclusion and exclusion criteria

This study strictly adhered to well-defined eligibility criteria. The scope encompassed research pertaining to “ferroptosis-TCM”, with specific attention given to original investigations and reviews issued in scientific journals using the English language. Excluded were dissertations, letters, commentaries, editorials, conference abstracts, and works with the same or similar titles published in different journals. The inclusion and exclusion criteria were finalized through discussions among team members and peer groups.

### 2.3 Statistical analysis

Research data were gathered from the WoSCC and processed using specific software tools: WPS Office 12.1.0 (Kingsoft Office, China) for data organization, VOSviewer 1.6.18 (Leiden University, Netherlands) and Pajek 64 5.16 (University of Ljubljana, Slovenia) for co-occurrence analysis, Citespace version 6.2.6R (developed by Chaomei Chen, China) for visual mapping, Scimago Graphica version 1.0.35 (https://www.graphica.app/, USA) for graphical analysis. Additionally, various R packages (R Studio, version 4.2.0) including chorddiag, enrichplot, ggplot2, and clusterprofiler were utilized for generating specialized graphics.

The chorddiag R package and VOSviewer were employed to generate maps depicting national/regional collaboration and publication analysis charts. For co-occurrence analysis involving institutions, authors, journal publications, keywords, and diseases, a combination of VOSviewer, Scimago Graphica, and Pajek was utilized. Citespace was used for visualized analysis and mapping of data related to countries/regions, institutions, authors, journals, co-citations, and keywords. In addition, we employed the clusterProfiler, enrichplot, and ggplot2 R packages to carry out enrichment analyses for Gene Ontology (GO) and Kyoto Encyclopedia of Genes and Genomes (KEGG) pathways.

Data regarding disease information were obtained from the Citexs Data Analysis Platform (https://www.citexs.com). This platform facilitates the generation of relevant visual graphs, enabling an in-depth analysis of the current state, key focus areas, and emerging trends within this field of study.

## 3 Results and discussion

Staying abreast of industry developments and comprehending the latest research findings in the current era of information proliferation pose escalating challenges. With the purpose of disseminating the contemporary state of research pertaining to “ferroptosis-TCM” on a global scale, encompassing data from 2012 to 2023, we have applied bibliometric analysis. This methodology provides a pioneering approach for the management and elucidation of knowledge frameworks within specific research domains (Wang C. et al., 2021; Chen et al., 2022; Han et al., 2022).

### 3.1 Annual output and trends

**FIGURE 1:**
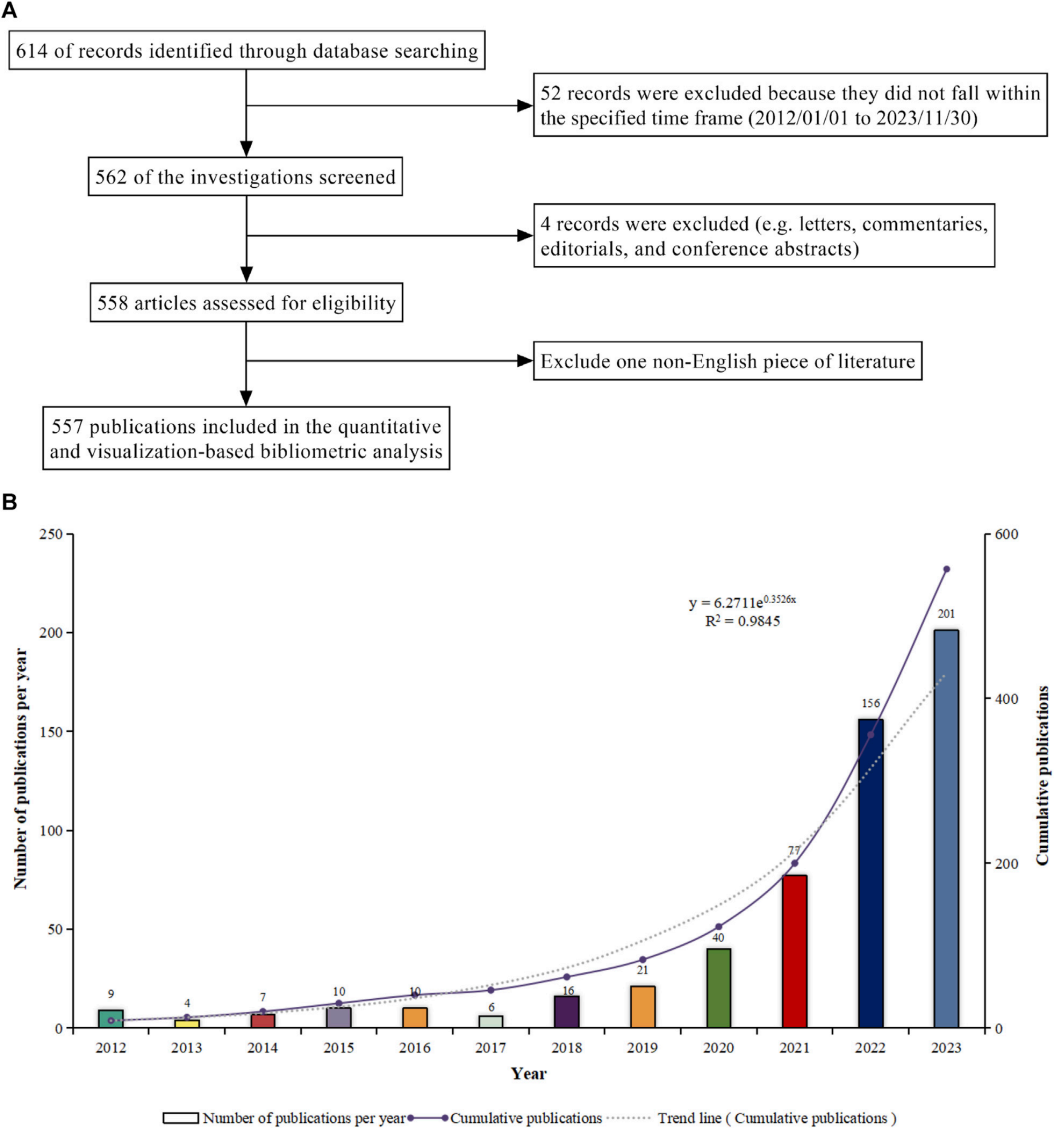
**(A)** Schematic representation of the literature search and selection process. **(B)** Trend analysis of research on “ferroptosis-traditional Chinese medicine” from 2012 to 2023.

Figure 1A depicts the procedure of data retrieval and collection. The quantity of scientific reports produced by a study within a given timeframe indicates its research progress (Koo, 2021; Li Z. et al., 2022; Sun G. et al., 2022). From 2012 to 2023, we compiled 557 relevant scientific reports on “ferroptosis-TCM”, comprising 458 original articles and 99 reviews, with an annual average of 50.64 publications. In the past 11 years, there has been a continuous increase in academic production within this domain. Commencing from 2021, the annual tally of pertinent publications surpassed 50, culminating at 201 in 2023. This surge stands for a more than 22-fold increase since 2012, with a yearly growth rate of 48.61%. This trend underscores the continuous rise in research activity and the significant importance of this field. An exponential equation (y = 6.2711e^0.3526x^, where x is the year and y the yearly publication count, R^2^ = 0.9845) was employed to accurately depict the annual pattern, resulting in a well-fitted curve (Figure 1B). This growth reflects a dynamic research landscape, contributing extensive knowledge that enriches both present and future studies. Consequently, we anticipate substantial advancements in “ferroptosis-TCM” research in the foreseeable future.

The rise in academic publications signifies a noteworthy inclination towards investigating the regulatory impacts of TCM on ferroptosis. This pattern underscores the swift expansion of this domain, drawing in a broader spectrum of researchers and accentuating its escalating prominence within the academic sphere. This anticipated expansion could also engender increased financial support, expertise, and attention from diverse stakeholders. Potential breakthroughs in understanding ferroptosis and its integration with TCM could have profound implications, such as the development of innovative treatments, diagnostic tools, or therapeutic strategies. Industry practitioners, including pharmaceutical companies and healthcare providers, should take note of the rising research activity in this area.

### 3.2 Global research landscape

**FIGURE 2:**
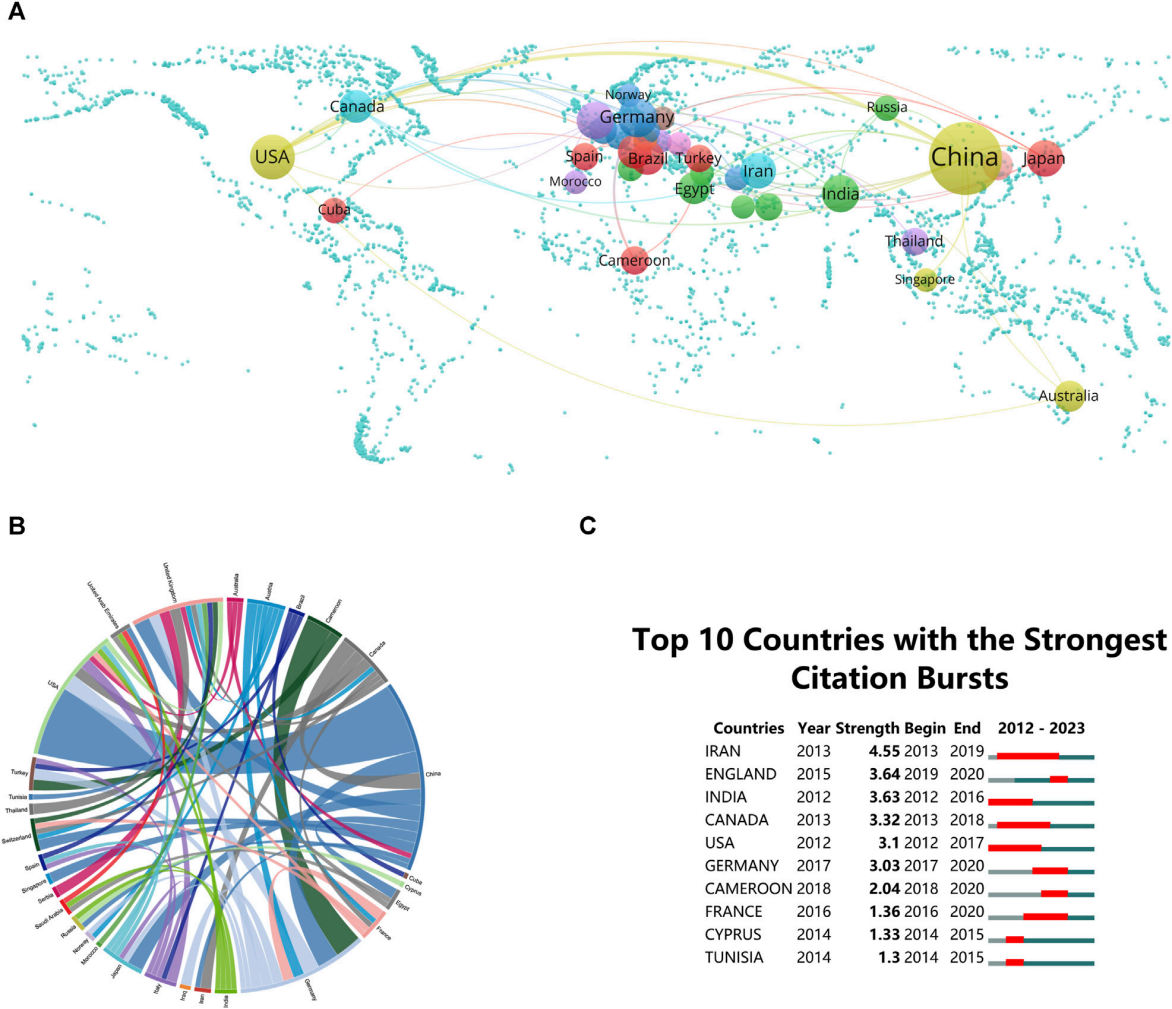
**(A)** Global distribution of “ferroptosis-traditional Chinese medicine (TCM)" research. **(B)** Chord diagrams illustrating international collaborations. **(C)** Research output on “Ferroptosis-TCM” from the top 10 countries (highlighted in red signifying increases in document production).

Global research on “ferroptosis-TCM” involves 57 countries/regions. To create maps of national collaboration networks, a minimum threshold of two publications per country/region was employed, as depicted in Figures 2A, B. Notably, China leads with 401 publications, constituting 71.99% of the total research output in this field. This highlights China’s significant role in advancing knowledge in this area. Following China, the USA and Germany contribute 7.18% (40 publications) and 3.77% (21 publications), respectively, to global research on “ferroptosis-TCM”, demonstrating the worldwide interest and involvement in this research.

The chord diagram utilizes peripheral curve segments to represent different countries and regions, with each segment’s length indicating the corresponding publication volume (Cao et al., 2023b). Moreover, the extent of connectivity among nations reflects the degree of their collaborative engagement. Notably, significant international collaboration was observed during the study period. The most frequent academic collaborations were observed between China and the USA, exhibiting a notable link strength of 13 (Figure 2B). The number of publications serves as an indicator of a nation or region’s prominence within this domain (Joshi, 2014). China and the USA, as leaders in publications related to “ferroptosis-TCM”, underscore their substantial academic influence. This robust collaboration has the potential to propel theoretical progress and tackle ongoing field-specific challenges (Dara et al., 2017). Such collaboration can facilitate the interchange of ideas, resources, and expertise, potentially accelerating theoretical advancements and addressing challenges in the field. Researchers and practitioners from different countries should consider leveraging these collaborations for mutual benefit.

Citation bursts play a vital role in the identification of publications that have witnessed notable surges in citations over a defined time frame (Cao et al., 2023b). These bursts shed light on the dynamic trends and directions within research fields. Analyzing publications experiencing rapid citation increases aids in identifying emerging trends within areas of high scholarly interest. Figure 2C illustrates the citation bursts for the leading 10 nations, with the red line denoting the burst magnitude for each country. During the period between 2013 and 2019, Iran witnessed a significant surge in publication citations (strength = 4.55), closely followed by England (strength = 3.64), indicating emerging trends and areas of high scholarly interest. Researchers and industry practitioners should pay attention to these countries and their specific research directions, as they may provide valuable insights and opportunities for further exploration and collaboration. Researchers and industry professionals can use this information to identify potential partners and track the evolution of research collaborations. This can help them gain a better understanding of the global distribution of expertise in the field.

Fundamentally, the worldwide dispersion, collaborative structures, and emerging directions within “ferroptosis-TCM” investigation imply a substantial shift towards comprehensive worldwide involvement. These discoveries underscore the necessity for expanded partnerships, pinpoint emerging research focal points, and signify prospects for researchers and industry stakeholders alike to leverage varied proficiency in promoting remedies for human ailments through TCM.

### 3.3 Institutional performance

**FIGURE 3:**
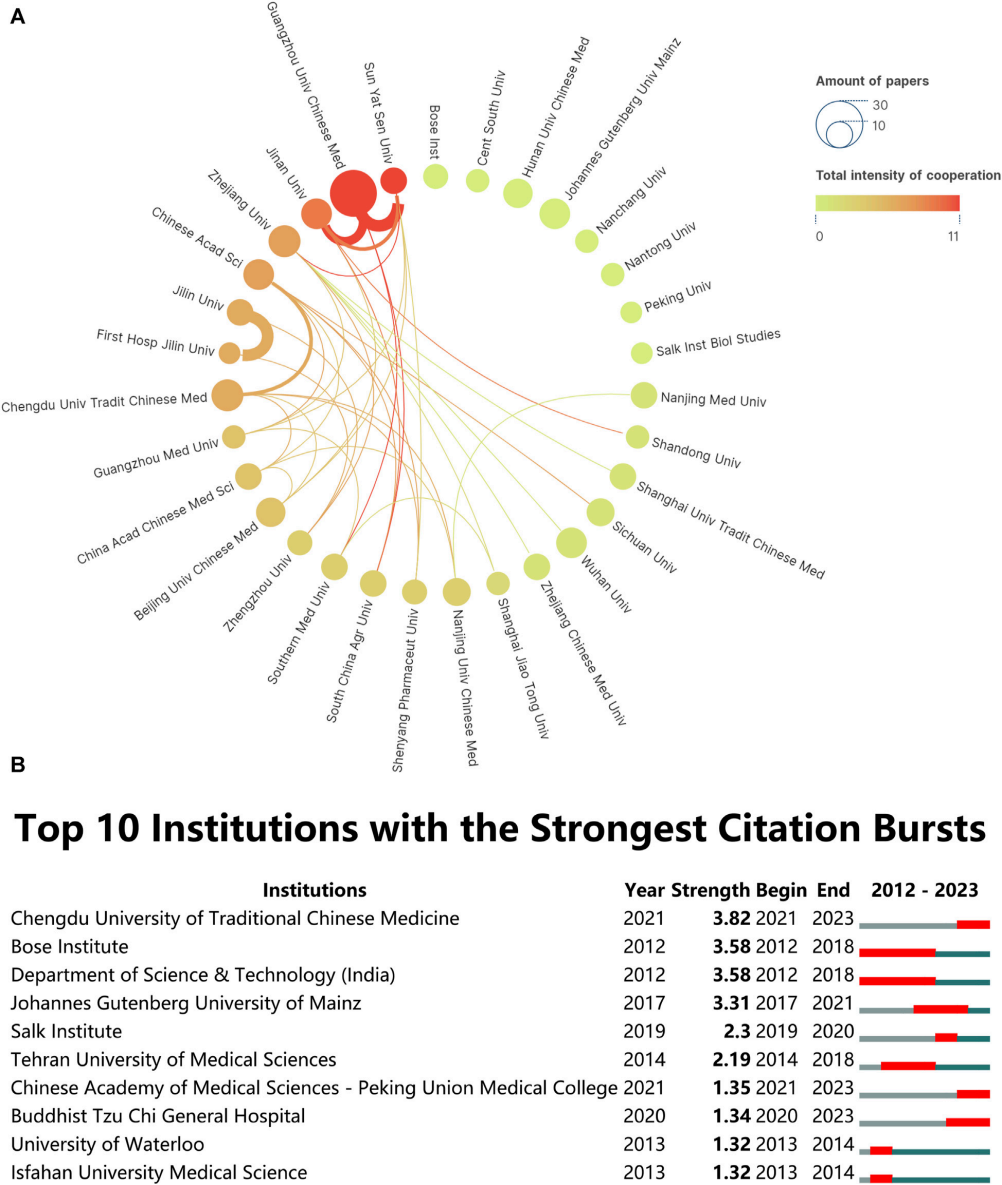
**(A)** Diagram illustrating the intensity of institutional cooperation. **(B)** Top 10 Institutions with Citation Bursts (Red Bars Represent Periods of Increased Citations).

Identifying top-performing institutions and analyzing their citation bursts provides valuable insights for researchers seeking collaborators and funding opportunities. The dynamic collaborative network among institutions signifies a vibrant research ecosystem. Over the past 11 years, global research on “ferroptosis-TCM” has made significant strides, involving over 760 entities. A collaboration network, illustrated in Figure 3A, was established among these institutions, requiring a minimum of six publications for inclusion. In this network, circles and texts represent each institution; connecting lines denote collaborative occurrences; line thickness signifies collaboration strength; gradient colors represent the total collaboration strength between an institution and others; circle size correlates with the institution’s publication count. Guangzhou University of Chinese Medicine emerges as the leading contributor, accounting for approximately 5.02% (28 publications) of the total research output. Following closely, Chengdu University of Traditional Chinese Medicine and Zhejiang University contribute 2.33% (13 publications) respectively. These institutions have demonstrated a substantial contribution to the field, making them potential collaborators for future research projects. Our analysis of interinstitutional collaboration revealed remarkable enthusiasm from Guangzhou University of Chinese Medicine and Sun Yat-sen University in engaging with partnering institutions. It is worth mentioning that the vast majority of these organizations prioritize domestic cooperation rather than international collaboration. The dynamic collaborative network among institutions indicates a vibrant research ecosystem in the “ferroptosis-TCM” field, which can foster innovation and knowledge exchange. This ecosystem provides fertile ground for sharing ideas, resources, and expertise. Researchers can tap into this ecosystem to stay updated on the latest developments, access shared resources, and enhance the overall quality of research.

Additionally, understanding which institutions are experiencing citation bursts can help researchers identify institutions with active and impactful research programs that may offer collaboration opportunities or funding prospects. When contemplating collaborations, one should evaluate not only the quantity of publications but also the enduring impact and flexibility of research endeavors over an extended duration (Cao et al., 2024). Using CiteSpace analysis (Figure 3B), this study identified institutions with significant citation bursts. Bose Institute and the Department of Science & Technology (India) experienced remarkably long burst periods in citations from 2012 to 2018. However, this trend declined in the last 5 years. In contrast, Chengdu University of Traditional Chinese Medicine exhibited a delayed citation burst from 2021 to 2023. This pattern suggests a shift in focus and a delayed timeline for research output in this institution’s field.

### 3.4 Author Contributions

**FIGURE 4:**
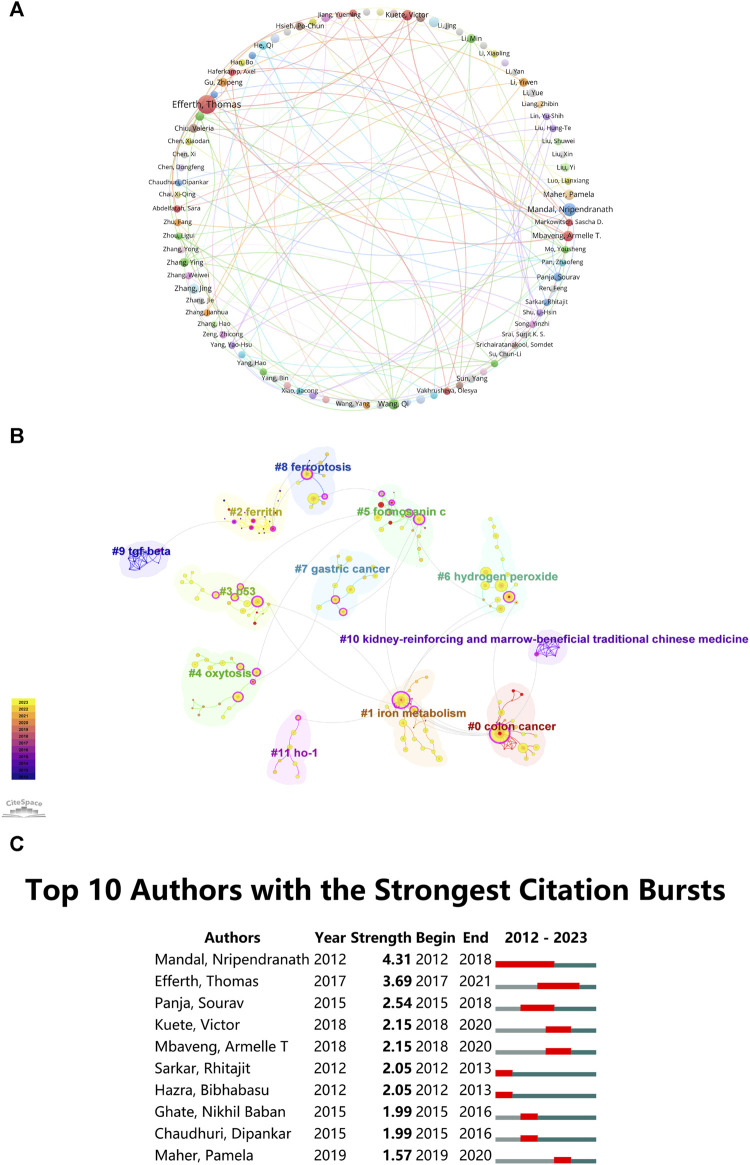
**(A)** Author Co-occurrence map. **(B)** Author cluster analysis. **(C)** Top 10 authors with significant citation bursts in “ferroptosis-traditional Chinese medicine” publications.

Recognizing prolific authors and analyzing their collaboration networks can help researchers identify key contributors in the field. Authors with high citation rates and a consistent publication history offer valuable insights and can guide future research directions. Following a thorough examination of authorship within the domain of ‘ferroptosis-based TCM’, a total of 3,615 authors emerged as significant contributors. Among these, eight authors were particularly prolific, each having authored a minimum of five papers. These authors likely possess in-depth knowledge and expertise in the field, making their work essential for those seeking guidance in “ferroptosis-TCM” research. To conduct a detailed examination of co-authorship networks, we utilized VOSviewer software to generate visualization maps. These maps were created with a baseline criterion of a minimum of three publications per author. In these representations, the dimensions of the circles are proportional to the number of publications by each author. Various color coding denotes discrete author clusters. The thickness of interconnecting lines among these circles indicates the degree of collaborative interactions. Remarkably, 90 authors exceeded this publication threshold, Thomas Efferth, Victor Kuete, and Armelle T. Mbaveng demonstrated the most robust collaborative relationships, as illustrated in Figure 4A. Furthermore, Thomas Efferth and Nripendranath Mandal were recognized as high-yielding scholars in the research on “ferroptosis-TCM”, emphasizing their essential contributions to this scientific field.

CiteSpace assesses network integrity and clustering clarity using Modularity (*Q* value) and Mean Silhouette (*S* value). A *Q* value above 0.3 indicates robust clustering, while an *S* value above 0.5 suggests clear and reasonable clustering (Wang B. et al., 2022). The study recorded a cluster modularity value (*Q*) of 0.8341 and a mean silhouette value (*S*) of 0.9492, indicating highly significant and clear delineation of keyword clusters. In Figure 4B, authors are categorized into 12 groups and labeled with keywords, including: #0 colon cancer, #1 iron metabolism, #2 ferritin, #3 p53, #4 oxytosis, #5 formosanin c, #6 hydrogen peroxide, #7 gastric cancer, #8 ferroptosis, #9 transforming growth factor-β (TGF-beta), #10 kidney-reinforcing and marrow-beneficial Traditional Chinese Medicine, #11 Heme Oxygenase-1 (HO-1).

Citation burst analysis serves as a critical metric, indicating the frequency of citations received by an author in a specific research domain during a defined timeframe (Jiang et al., 2022; Li and Du, 2022). Figure 4C presents the top ten authors with the highest citation counts in the realm of “ferroptosis-TCM”. Nripendranath Mandal leads with a burst strength of 4.31, closely followed by Thomas Efferth and Sourav Panja. Researchers and industry practitioners can refer to their work as a reference framework for future research directions, ensuring that their research builds upon the foundation of influential and highly cited studies. These influential authors offer valuable guidance for researchers navigating this research domain. Moreover, their work can inform industry practitioners about key players and emerging trends, potentially influencing their strategic decisions and collaborations.

### 3.5 Analysis of high-contributing journals

**FIGURE 5:**
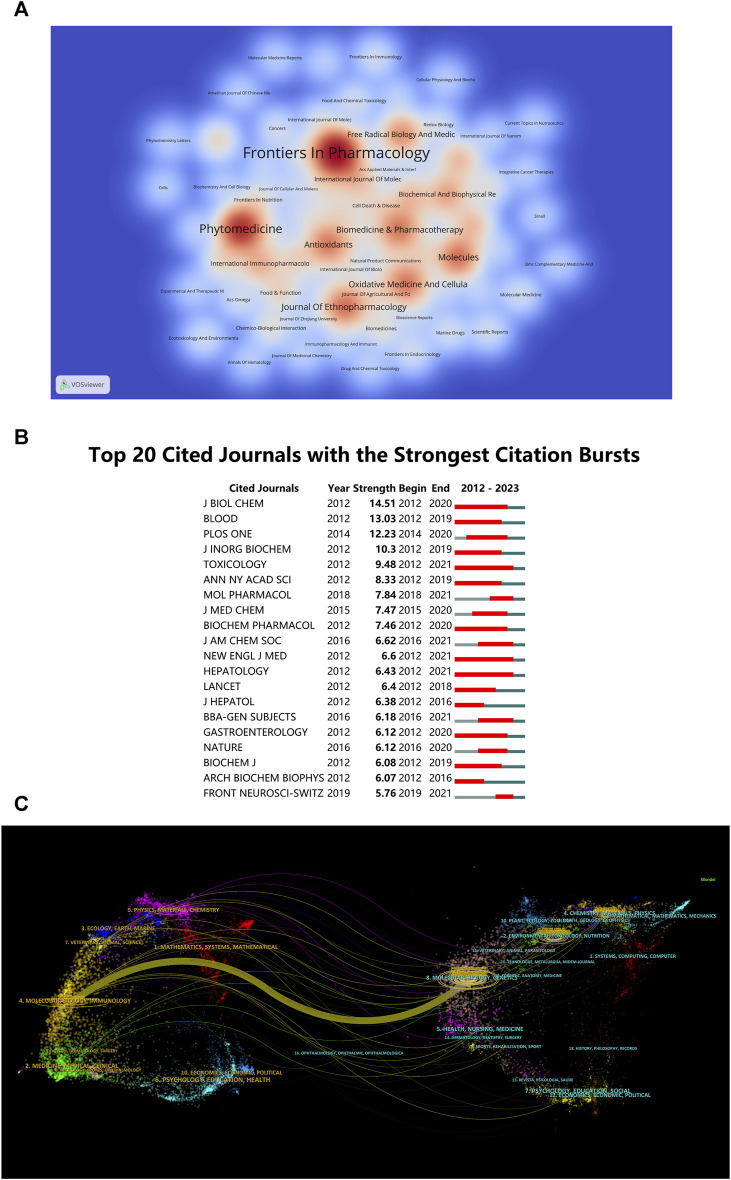
**(A)** Density visualization map of Journal citations. **(B)** Top 20 journals with significant citation bursts. **(C)** Dual-map overlay of journals in “ferroptosis-traditional Chinese medicine” research.

Drawing from visualized journal publication data, it becomes evident that 268 journals have disseminated articles related to “ferroptosis-TCM”. To illustrate the dispersion of these documents among journals, a thermodynamic chart, as depicted in Figure 5A, has been constructed, employing a minimum criterion of two documents per journal. The intensity of color in the chart corresponds to the quantity of journal papers issued. The journal ‘*Frontiers in Pharmacology*’ ranks highest in the number of published documents (n = 33, 5.92%), followed by ‘*Phytomedicine*’ (n = 24, 4.31%), ‘*Journal of Ethnopharmacology*’, and ‘*Molecules*’ (n = 14, 2.51%). This provides researchers with a comprehensive understanding of the publication landscape in this field, helping them select appropriate journals for their own work and ensuring that their work reaches the relevant audience. Additionally, Figure 5B lists the top 20 journals with the strongest citation bursts for “ferroptosis-TCM” publications. Researchers can assess the significance of specific journals and prioritize them when citing or referring to previous research in their own work.

The dual-map overlay technique effectively presents the interdisciplinary dispersion of journals, the development of citation trajectories, and the relocation of scientific research centers (Yuan et al., 2023). Labels on the map depict the various subject areas covered by the journals. Journals referencing others are positioned on the left side, while those being referenced are situated on the right side (Zhang T. et al., 2022). Diverse colored lines visually illustrate the citation pathways, originating from the citation map and concluding there. The breadth of these connecting pathways is closely associated with the frequency of *z*-score-scaled citations. Figure 5C depicts the classification of research pertaining to “ferroptosis-TCM” into three key domains: molecular biology, immunology, and genetics. Such interdisciplinary insights can inspire collaboration among researchers from diverse backgrounds, fostering the exchange of ideas and innovative approaches. The findings can assist industry professionals, including pharmaceutical companies and biotechnology firms, in pinpointing potential investment areas and opportunities for product development in the “ferroptosis-TCM” domain. Understanding the interdisciplinary nature of research can guide them in recruiting experts from relevant domains and developing innovative therapies or products related to ferroptosis and TCM.

### 3.6 Co-cited references

**FIGURE 6:**
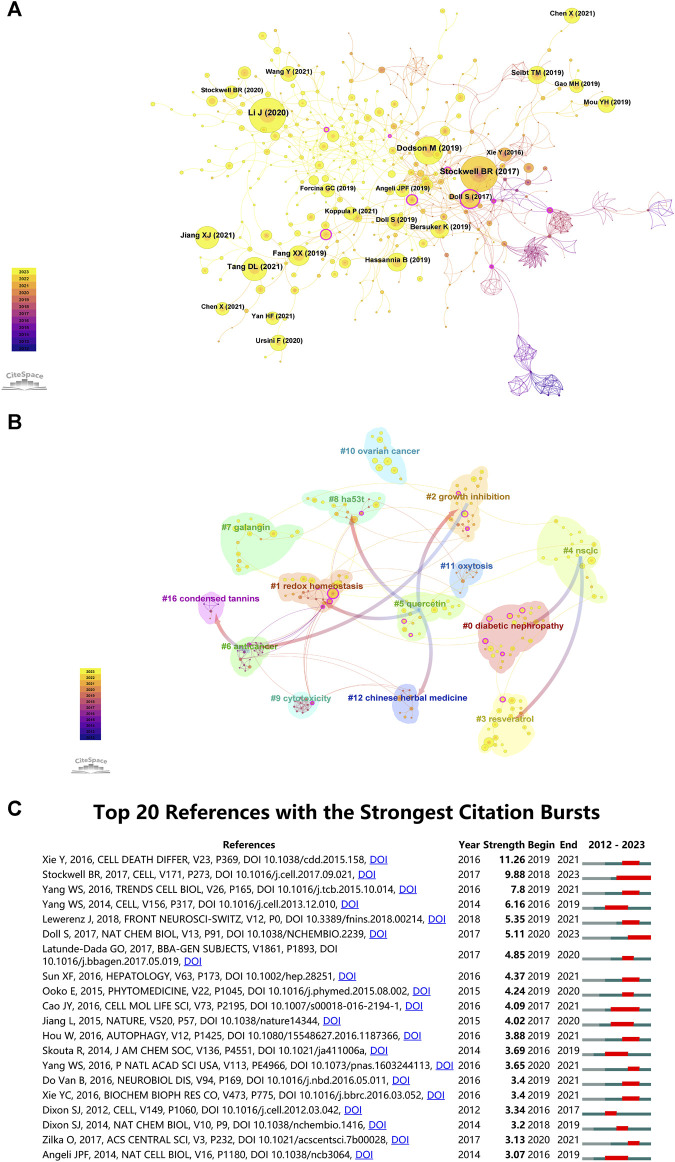
**(A)** Co-citation analysis chart for “ferroptosis-traditional Chinese medicine”. **(B)** Co-cited literature network map. **(C)** Top 20 references with the highest citation bursts.

Understanding which works have garnered the most attention and citations is essential for researchers as it helps them build upon existing knowledge and concepts, contributing to the advancement of their respective fields. Figure 6A displays a co-citation network diagram, delineating the co-citation network of literature related to “ferroptosis-TCM” from 1 January 2012, to 30 November 2023, as analyzed using CiteSpace. In this representation, the sizes of the spheres, aggregating their sizes across annual rings, are proportionate to their co-citation frequencies. The color scheme spans from purple, indicating earlier citation instances, to yellow, representing more recent citations. Spheres with overlapping colors indicate sustained citation over the specified years. The interconnecting lines between spheres illustrate the co-citation relationships among various pieces of literature. Notably, nodes highlighted in magenta signify pivotal nodes in the network, characterized by a centrality exceeding 0.1. The review titled ‘Ferroptosis: A Regulated Cell Death Nexus Linking Metabolism, Redox Biology, and Disease’, authored by Brent Stockwell et al. and published in *Cell* in 2017, stands out among the most frequently cited works, with the highest co-citation count (n = 60) (Stockwell et al., 2017). The 2020 *Cell Death & Disease* paper ‘Ferroptosis: past, present and future’ by Li Jie et al. followed with 59 co-citations (Li et al., 2020).

CiteSpace employs metrics such as Modularity (*Q* value) and Mean Silhouette (*S* value) to assess network structures and clustering clarity. A *Q* value above 0.3 indicates significant clustering, while an *S* value higher than 0.5 indicates clear and effective clustering. In the analysis conducted, the computed values were *Q* = 0.8569 and *S* = 0.9535, confirming the presence of robust and convincing clustering structures within the network. This result emphasizes the reliability of the employed citation clustering method. The analysis identified 13 distinct clusters, labeled as #0 diabetic nephropathy, #1 redox homeostasis, #2 growth inhibition, #3 resveratrol, #4 non-small cell lung cancer, #6 anticancer, #7 galangin, #8 ha53t, #9 cytotoxicity, #10 ovarian cancer, #11 oxytosis, #12 Chinese herbal medicine, #16 condensed tannins, as illustrated in Figure 6B. Researchers can use this information to ensure their work aligns with established research clusters, increasing the likelihood of their research being recognized and cited by peers and experts.

The analytical capabilities of CiteSpace were used to detect citation bursts, highlighting studies that have garnered significant scholarly attention in the field of “ferroptosis-TCM”. As depicted in Figure 6C, an analysis of the top 20 references reveals their substantial impact, evidenced by significant citation bursts. The field experienced a notable surge in citations starting from 2016, with numerous co-citation references gaining substantial citations in the following years. This trend highlights the lasting significance of research in “ferroptosis-TCM”. Notably, in 2019, 45% (9 out of 20) of these references exhibited citation bursts, marking it as the year with the highest frequency, followed by 2016 and 2020, accounting for 20% (4 out of 20) and 15% (3 out of 20) of the bursts, respectively. The study with the most prominent citation burst (strength = 11.26) was titled ‘Ferroptosis: Process and Function’, originally published in *Cell Death & Differentiation* (Xie et al., 2016). This was closely followed in impact by the works of Brent Stockwell et al. (Stockwell et al., 2017), and Wan Seok Yang et al. (Yang and Stockwell, 2016). Researchers can identify key studies that have gained significant scholarly attention and understand when these bursts of interest occurred. This information can help researchers stay updated on emerging trends and focus their efforts on areas of high impact. By analyzing co-citation references and identifying works that are frequently cited together, researchers can identify potential collaborators who share common research interests, enhancing their ability to conduct collaborative research projects. Collaborating with authors whose works are frequently cited alongside one’s own research can enhance the visibility and impact of their work. Industry practitioners can use the information on influential works and research trends to inform their strategic decisions, such as identifying research partnerships or investment opportunities in areas aligned with the most cited and impactful studies.

### 3.7 Keyword analysis

**FIGURE 7:**
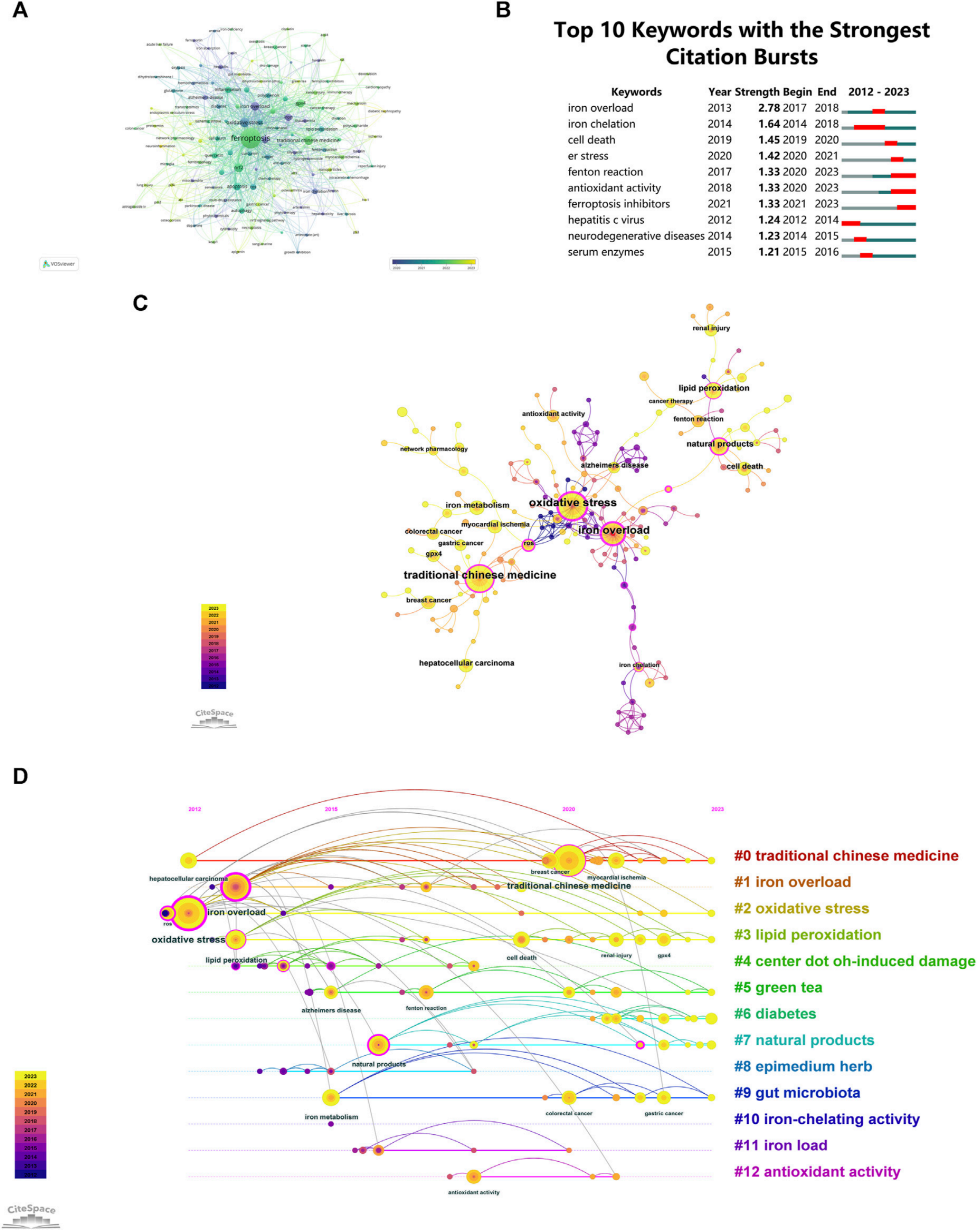
**(A)** Keywords intensity visualization timing overlay. **(B)** Top 10 keywords with significant citation bursts. **(C)** Co-occurrence analysis chart of Keyword frequencies. **(D)** temporal trends in Keyword Co-occurrence.

Keyword co-occurrence clustering analysis was performed using VOSviewer software, with a minimum keyword occurrence threshold set at three instances. Keywords meeting these criteria were included in the graph. A total of 131 keywords were selected from the initial pool of 1,469 keywords (after removing duplicates) to create the visualized network. Figure 7A represents a word frequency-time graph, where circles and corresponding labels collectively constitute a node. The size of each circle corresponds to the frequency of keyword occurrence, while the thickness of connections between circles indicates the strength of relationships among keywords. The color of each circle, as indicated in the lower right corner, gradually changes to represent the average year of appearance. Blue indicates earlier appearances, while yellow represents later appearances of the keywords. Based on the data presented in Figure 7A, it is evident that ferritin, phytochemicals, and thalassemia were topics of earlier research, while metabolomics, neuroinflammation, and diabetic nephropathy have emerged as recent research hotspots and directions. This information is invaluable for researchers as it highlights areas of growing interest and potential for further exploration.

The identification of keyword bursts, particularly those with substantial increases in citations, indicates areas of intense scholarly attention (Figure 7B). Figure 7B illustrates that 30% of the keywords (3 out of 10) experienced their initial citation surge in 2020, followed closely by 2014, which accounted for 20% of the surges (2 out of 10). Notably, 40% of these keywords maintained elevated citation rates over the past 3 years, indicating a sustained and growing interest in specific research domains. The keyword ‘iron overload’ displayed the most significant burst, with a burstness value of 2.78, followed by ‘iron chelation’ and ‘cell death’, with burstness values of 1.64 and 1.45, respectively. These insights bear significant implications for researchers in their respective fields.

Co-occurrence analysis reveals the relationships between keywords and identifies key themes and concepts in the field. Figure 7C illustrates a co-occurrence analysis chart of keyword frequencies, utilizing CiteSpace to analyze the co-occurrence of “ferroptosis-TCM” from 1 January 2012, to 30 November 2023. The spheres’ sizes indicate the cumulative size of spheres on yearly rings, in proportion to the frequency of keyword usage. Purple represents earlier keyword appearances, while yellow indicates later appearances, and overlapping colors indicate citations in corresponding years. Lines connecting spheres represent co-citation connections among literature, and nodes highlighted in magenta represent critical nodes with a centrality above 0.1. Figure 7C shows that the most frequently co-occurring keyword is ‘oxidative stress’, followed by ‘iron overload’ and ‘traditional Chinese medicine’, which are tied for second place.

Understanding the evolving trends and hotspots in “ferroptosis-TCM” research is crucial as it can guide investment and collaboration decisions, ensuring strategic alignment with current research interests. Figure 7D depicts a temporal analysis of keyword frequency clustering within hotspots. The diagram showcases circles of varied sizes, where the cumulative diameter on each annual ring corresponds to the keyword frequency. Interconnections among keywords signify their co-occurrence. The color scheme is informative: purple represents earlier keyword emergence, yellow denotes recent appearances, and overlapping colors indicate consistent keyword presence across years. Magenta nodes highlight keywords with significant centrality, underscoring their pivotal role as network hubs. Keywords within the same cluster are horizontally aligned. The timeline orientation positions the initial keyword occurrence at the top, progressing chronologically to the right. This visualization aids in understanding keyword distribution within each cluster, with a larger quantity signifying greater cluster relevance. Additionally, it outlines the temporal duration of keywords in each cluster. The keywords are categorized into 13 distinct groups: #0 Traditional Chinese Medicine, #1 Iron Overload, #2 Oxidative Stress, #3 Lipid Peroxidation, #4 •OH-induced Damage, #5 Green Tea, #6 Diabetes, #7 Natural Products, #8 Epimedium Herb, #9 Gut Microbiota, #10 Iron-Chelating Activity, #11 Iron Load, #12 Antioxidant Activity. These clusters encapsulate primary themes and hotspots in the research domain, reflecting their significance in scientific advancements, therapeutic potential, or clinical applicability. Researchers and industry practitioners can use this information to navigate the various research directions and pinpoint areas of particular relevance, helping them focus their efforts more effectively.

It is not difficult to observe that within the multiple analyses of keywords above, ‘iron overload’ and ‘oxidative stress’ appear with the highest frequency. TCMs offer a plethora of natural antioxidant substances, encompassing polyphenols, alkaloids, flavonoids, terpenoids, etc., all of which exhibit excellent antioxidant properties. Therefore, future investigations should explore the potential of TCMs in modulating iron metabolism in the context of oxidative stress, aiming to identify potential targets for synergistic combination therapy, with the goal of achieving a “one TCM-multiple targets " approach for future interventions. To date, progress has already been made in this area with compounds such as baicalein, curcumin, and epigallocatechin gallate (Mandel et al., 2008; Perez et al., 2009; Dijiong et al., 2019; Guerrero-Hue et al., 2019; Kose et al., 2019; Rainey et al., 2019).

### 3.8 Key genes and pathways

**FIGURE 8:**
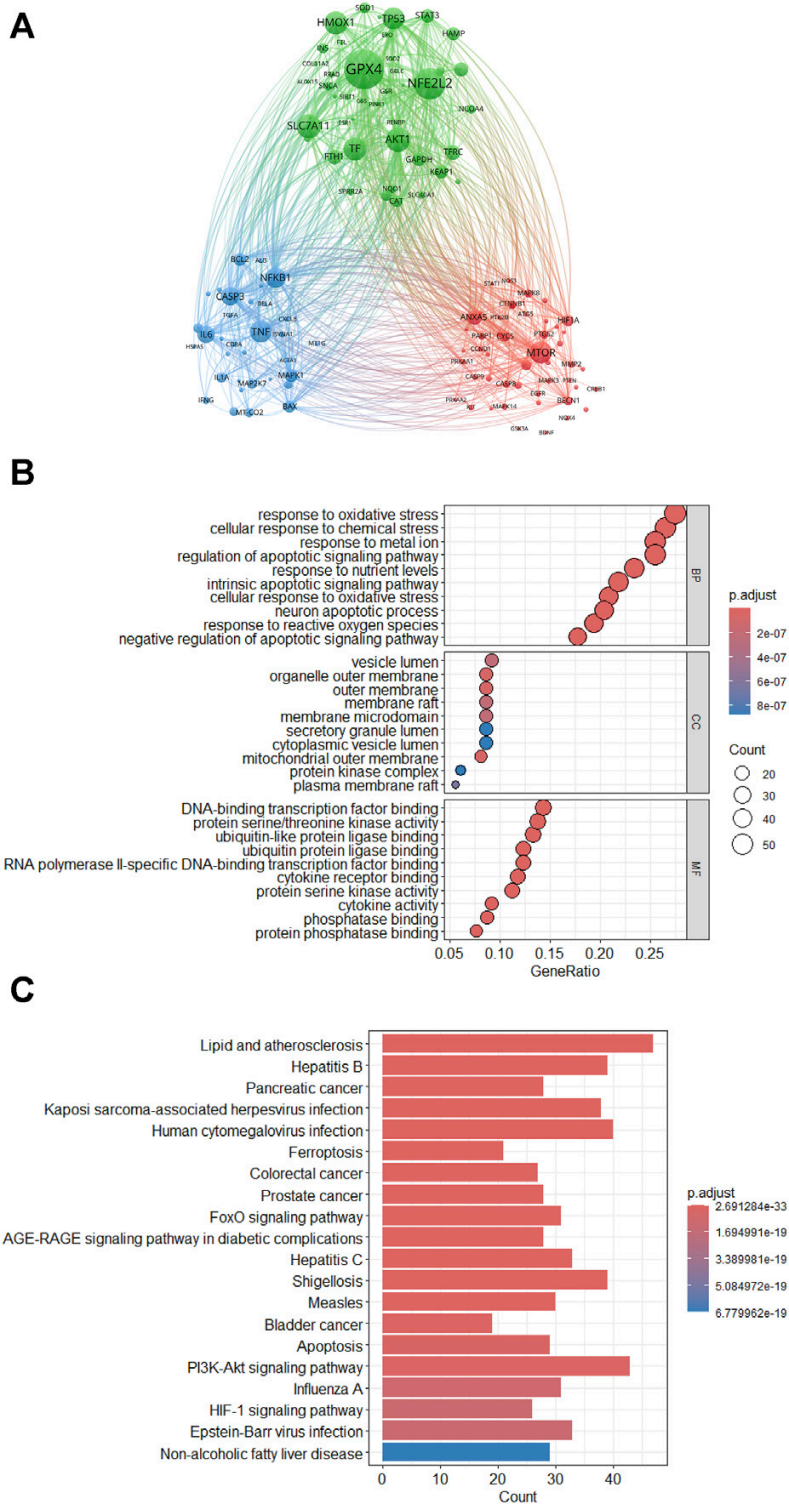
**(A)** VOSviewer critical gene clustering visualization. **(B)** Bubble Plots of gene Ontology enrichment analysis. **(C)** Kyoto Encyclopedia of genes and Genomes pathway enrichment analysis.

Utilizing VOSviewer software, we conducted a co-occurrence clustering analysis of genes related to “ferroptosis-TCM”. From a dataset of 557 articles obtained from the Citexs big data platform, we extracted a total of 1,109 genes, each appearing at least 6 times, which was then visualized in a map (Figure 8A). This visualization is categorized based on gene function similarity or their co-occurrence in research. Every node consists of a circle and a label, where the circle’s size corresponds to the gene’s frequency of occurrence, and the line thickness connecting circles indicates the strength of gene associations. Nodes of distinct colors form separate clusters, with each color symbolizing genes grouped within different domains, signifying particular biological or medical gene categories. These clusters represent specific biological or medical gene groups, enabling researchers to identify key genes and their functions within the context of ferroptosis and TCM.

Genes associated with “ferroptosis-TCM” form the core components of this research focus, effectively acting as dual indicators: one for recognizing shifting research patterns and the other for identifying changes in research orientation and the deepening of investigations. Illustrated in Figure 8A, the top three genes with the highest frequency were GPx4, NFE2L2, and SLC7A11. The sensitivity of ferroptosis correlates directly with the expression level of nuclear factor erythroid 2 like 2 (Nrf2), also known as nuclear factor erythroid 2-related factor 2 (NFE2L2). An increase in Nrf2 expression hinders ferroptosis, while a decrease in expression promotes it (Sun et al., 2016; Dodson et al., 2019). The mechanisms through which Nrf2 regulates ferroptosis encompass two primary aspects. Firstly, Nrf2 amplifies the antioxidant system by upregulating the expression of GSH and glutathione peroxidase 4 (GPx4), thereby enhancing antioxidant functionality. Secondly, Nrf2 stimulates the expression of ferritin and ferroportin, aiding in the storage or export of free iron. Consequently, this reduction in iron accumulation averts ferroptosis (Yang et al., 2017). Solute carrier family 7 member 11 (SLC7A11) functions as a cystine/glutamate antiporter (System Xc^−^), playing a crucial role in importing cystine for GSH biosynthesis and antioxidant defense (Koppula et al., 2021). In response, employing TCMs to target the specified pivotal genes for modulating ferroptosis could potentially offer an innovative strategy for preventing and managing human illnesses. This encompasses, without restriction, enhancers of iron metabolism, controllers of Nrf2-associated routes, and substances that inhibit or activate system Xc^−^ and GPx4.

Moreover, we performed GO and KEGG enrichment assessments on genes linked to “ferroptosis-TCM”, with a focus on genes referenced in the articles two or more times (Figures 8B,C). Researchers can use this information to pinpoint the specific functions and pathways influenced by these genes, guiding their experimental design and hypothesis generation. The identification of key genes and pathways can also facilitate collaborations between researchers and industry professionals, as it allows for the targeted leveraging of academic expertise in specific genes and pathways, potentially accelerating the development of novel treatments or interventions. The bubble chart for GO enrichment analysis illustrates each bubble as a GO term. Bubble size corresponds to the quantity of genes linked to that function, while color indicates the level of enrichment. The X-axis displays the GeneRatio, signifying the proportion of genes associated with the GO term in relation to the total gene count in the background gene set. A higher GeneRatio suggests a greater number of genes associated with the GO term, potentially indicating greater significance. The Y-axis categorizes GO terms into various biological processes, molecular functions, or cellular components.

Figure 8B illustrates the GO functional enrichment results, covering biological processes (BP), molecular functions (MF), and cellular components (CC). In terms of BP, the genes display enrichment in functions associated with responses to oxidative stress and cellular responses to chemical stress. Regarding CC, the genes exhibit enrichment in functions related to the vesicle lumen, outer membrane, and organelle outer membrane. Concerning MF, the genes show enrichment in functions such as DNA-binding transcription factor binding and protein serine/threonine kinase activity. Identifying specific cellular components and molecular functions enriched in these genes can assist researchers in pinpointing the organelles and activities critical to ferroptosis, thus facilitating the development of targeted interventions or therapies.

Furthermore, we performed KEGG pathway enrichment analysis to identify the top 10 signaling pathways, which is presented as a histogram. The X-axis represents the number of genes significantly enriched in each pathway, while the Y-axis displays different signaling pathways. The height of each column in the histogram signifies the gene count within the pathway and its level of significance after enrichment. As shown in Figure 8C, this research area is predominantly linked to signaling pathways such as ‘Lipid and Atherosclerosis’ and ‘PI3K/AKT signaling pathway’. This information can be of particular interest to industry practitioners, including pharmaceutical companies, as it suggests potential therapeutic targets or areas for drug development. Understanding the signaling pathways enriched in this context can guide industry professionals in designing interventions or treatments related to ferroptosis and its implications for various health conditions.

In relation to signaling pathways, as illustrated in Figure 8C, this subject is primarily associated with pathways such as lipid metabolism and atherosclerosis. It is noteworthy that various age-related diseases are frequently observed in the middle-aged and elderly population. Furthermore, these individuals are susceptible to concurrent chronic conditions, including cardiovascular and cerebrovascular diseases (atherosclerosis). To comprehensively grasp the implications of these changes in the context of a globally aging population, it is imperative to explore the capacity of TCMs in modulating ferroptosis and the aforementioned signaling pathways. The ultimate objective is to identify potential targets for a synergistic combination therapy.

### 3.9 Related diseases

**FIGURE 9:**
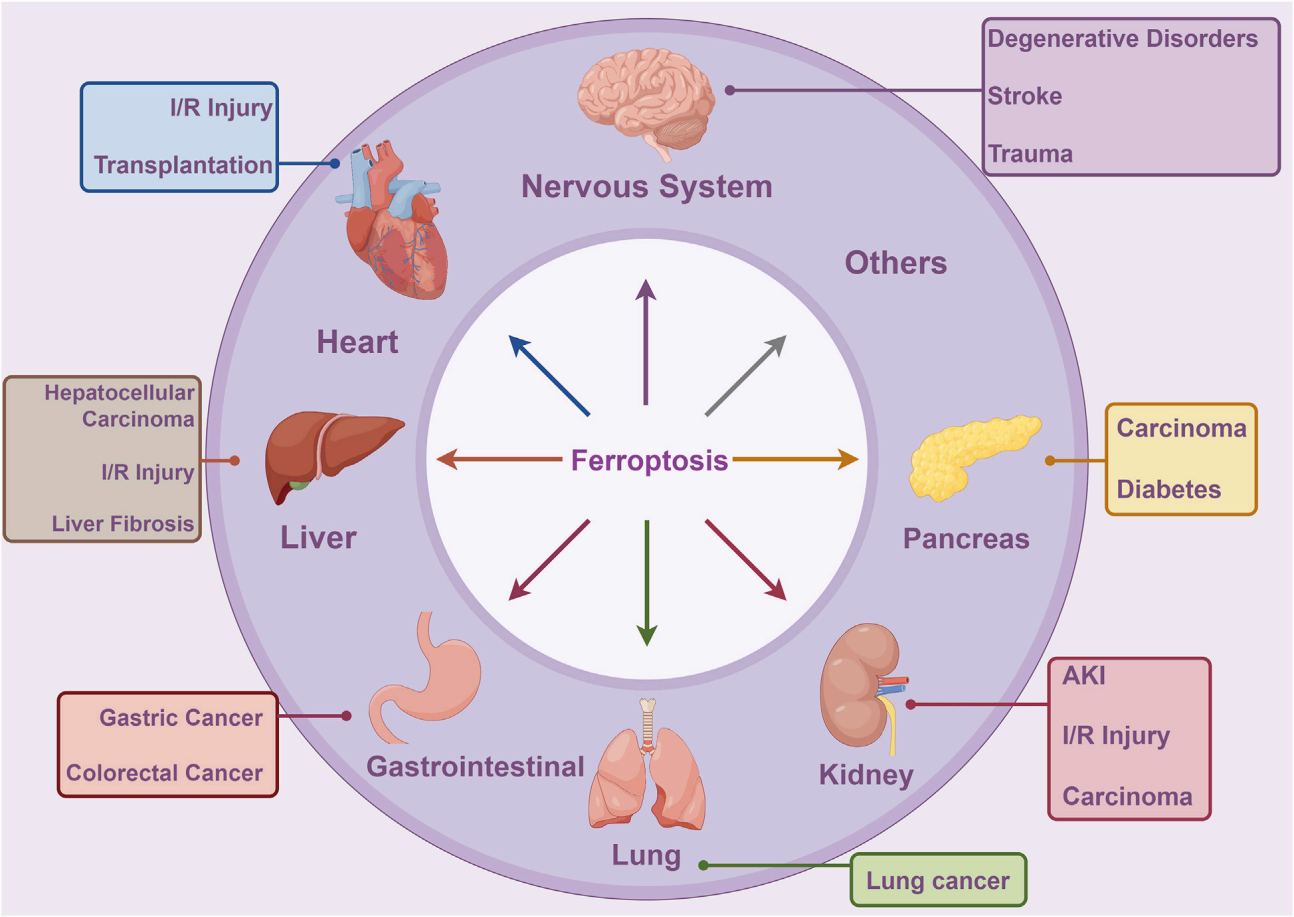
Ferroptosis plays significant roles in various systemic disorders, including conditions affecting the nervous system, cardiovascular diseases, hepatic disorders, gastrointestinal ailments, respiratory illnesses, renal conditions, pancreatic maladies, and others. This figure was created using Figdraw (https://www.figdraw.com/static/index.html#/). Abbreviations: AKI, acute kidney injury; I/R Injury, ischemia/reperfusion injury.

**FIGURE 10:**
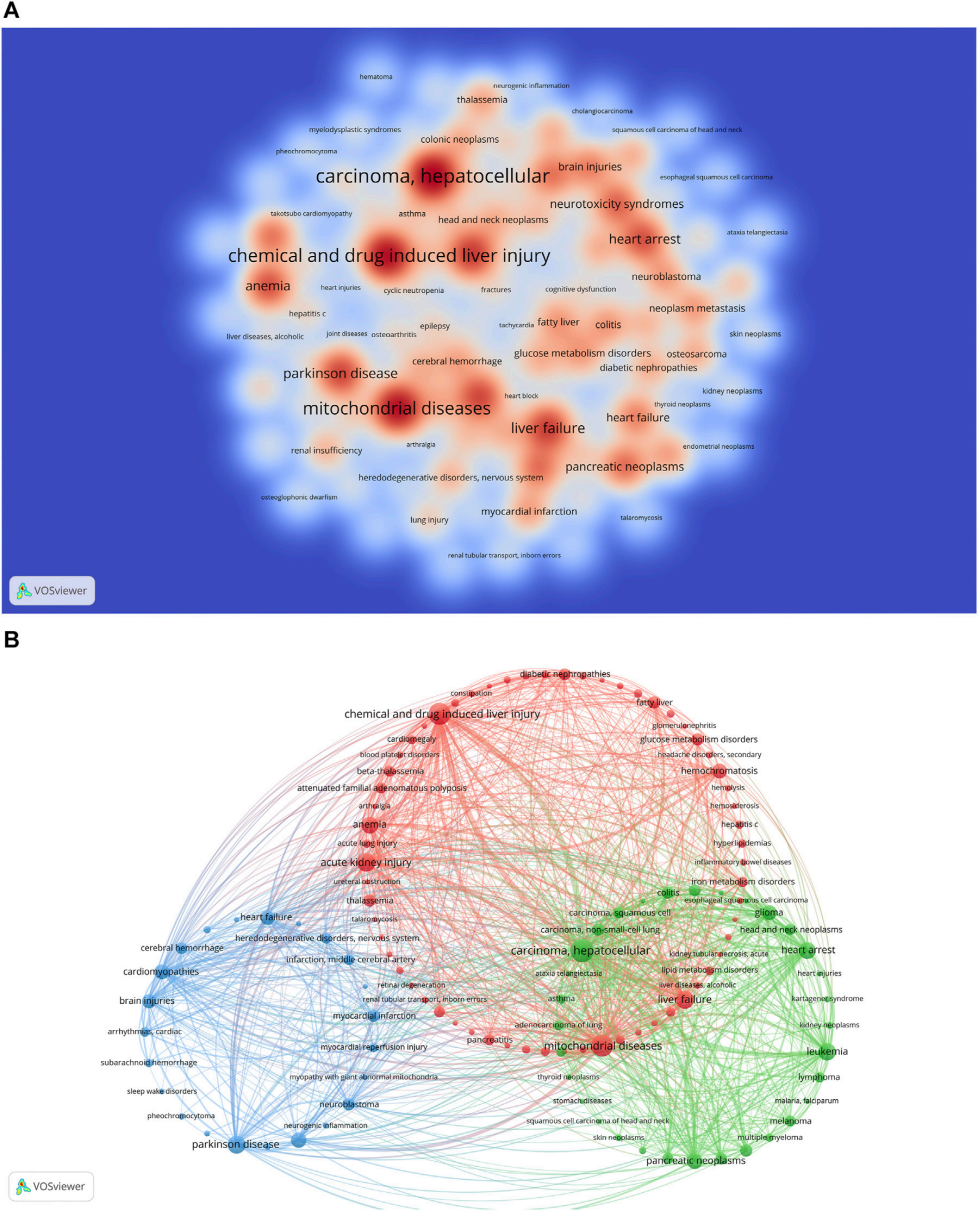
**(A)** Density visualization map of related diseases. **(B)** Disease clustering analysis chart.

In recent investigations, it has been discovered that the regulatory role of ferroptosis is pivotal in the initiation and advancement of various illnesses. This has positioned ferroptosis as a central and extensively researched area, aiming to improve the management and prognostication of associated conditions (Figure 9) (Li et al., 2020; Stockwell, 2022; Wang X. et al., 2023). Understanding the diseases most closely associated with “ferroptosis-TCM” research allows researchers to focus their efforts on specific medical conditions. This targeted approach can accelerate the development of drugs and therapies tailored to address these diseases, ultimately increasing the potential for more effective treatments. The Citexs Data Platform identified 765 diseases from 557 articles, with a minimum requirement of five articles mentioning each disease for inclusion. These diseases that met the criterion were visualized in a heatmap, created using VOSviewer, illustrating the frequency or relationships of diseases related to “ferroptosis-TCM” research (Figure 10A). The top five most frequently mentioned diseases include hepatocellular carcinoma, chemical and drug-induced liver injury, mitochondrial diseases, acute kidney injury, and liver failure. Additionally, a co-occurrence cluster analysis was performed, also requiring a minimum of five occurrences for each disease, using VOSviewer (Figure 10B). In this visualization, nodes—represented by circles and labels—are sized according to disease frequency. The thickness of lines connecting circles indicates the strength of relationships between diseases, and various colors represent distinct clusters corresponding to specific disease categories.

The identification of diseases associated with “ferroptosis-TCM” research can also stimulate efforts to discover biomarkers. Researchers can explore potential biomarkers associated with both ferroptosis and these specific diseases, which may have implications for diagnosis, prognosis, or therapy. In summary, these findings provide valuable insights into the diseases most closely linked to “ferroptosis-TCM” research, guiding researchers and industry practitioners toward targeted research, drug development, interdisciplinary collaborations, and potential clinical applications. Understanding the disease landscape in this context can pave the way for advancements in the treatment and management of related medical conditions.

### 3.10 TCM intervention of ferroptosis based on Yin-Yang theory

TCM modulates ferroptosis by promoting it in tumor cells (addressing excess) and inhibiting it in other diseases (addressing deficiencies), aligning with the treatment principle of balancing Yin and Yang.

#### 3.10.1 Damaging the excess

Disruptions in the signaling pathways associated with PCD may result in uncontrolled cell proliferation and, ultimately, cancer (Refaat et al., 2014). This leads to abnormal cell proliferation (excess Yang) and a significant reduction in PCD (deficiency in Yin). The treatment approach for cancer involves promoting ferroptosis (nourishing Yin) in tumor cells to inhibit their abnormal proliferation (restricting Yang) (Wang et al., 2023b), as stated in the Huang Di Nei Jing (Huangdi’s Internal Classic): “When Yang is excessive, treat with Yin; when Yin is deficient, treat with Yang”. TCM triggering ferroptosis primarily focuses on tumor-related research, encompassing various cancers such as lung (Han et al., 2023), breast (Li et al., 2023), stomach (Huang et al., 2024), liver (Wu et al., 2022), and ovarian (Chan et al., 2020) cancer, etc. The intervention mechanisms include Chinese herbal compounds, monomers, with key pathways involving the GPx4/GSH pathway, iron metabolism pathway, lipid metabolism pathway, and others (Wang et al., 2023b).

#### 3.10.2 Replenishing the deficiencies

In contrast to carcinomas, diseases such as acute kidney injury (Shi et al., 2023), type 2 diabetes mellitus (Miao et al., 2023), non-alcoholic fatty liver disease (Wang S. et al., 2022), and age-related orthopaedic diseases (Ru et al., 2023) often involve a decline in the function of tissues and organs. This is characterized by reduced normal cell proliferation (Yang deficiency) and an increase in PCD processes such as aging, apoptosis, and necrosis (excessive Yin). The approach to addressing these diseases includes replenishing deficiencies by inhibiting ferroptosis in normal cells (restricting Yin) to maintain both their quantity and functional integrity (nourishing Yang), as prescribed in the Huang Di Nei Jing (Huangdi’s Internal Classic): “When Yin is excessive, treat with Yang; when Yang is deficient, treat with Yin”. TCM inhibiting ferroptosis can intervene in various diseases or pathological conditions such as cerebral infarction (Hui et al., 2022), atherosclerosis (Zhang J. et al., 2023), Parkinson’s Disease (Wu et al., 2021), among others. Chinese medicine suppresses cell ferroptosis through herbal compounds, monomers, and external treatments (acupuncture and moxibustion) (Chen et al., 2024).

### 3.11 Challenges and future vistas

Despite significant advancements in “ferroptosis-TCM” research, ongoing critical challenges demand immediate attention.

#### 3.11.1 Achilles’ heel of present ferroptosis-related research

As of now, the majority of *in vivo* investigations pertaining to ferroptosis have been dependent upon preclinical animal models (Sun et al., 2023). However, there are several obstacles that hinder their transition into clinical use. Research and clinical trials focused on the molecular facets of ferroptosis have barely delved beneath the surface of this PCD pathway. In fundamental research, the central inquiry revolves around identifying the ultimate executor responsible for triggering ferroptosis after lipid peroxidation. Such a discovery may also reveal additional distinguishing characteristics of ferroptosis, setting it apart from other forms of PCD. While ferroptosis possesses unique features that differentiate it from other PCD modalities, recent studies have raised questions about its distinctiveness. These studies suggest interactions between ferroptotic factors and components associated with other PCD pathways, such as autophagy and pyroptosis (Ru et al., 2023). A comprehensive understanding of the interplay within the ferroptosis system, rather than focusing solely on individual regulators, is crucial for gaining deeper insights into the mechanisms of ferroptosis. As exploration delves deeper, this crosstalk may yield novel insights and offer opportunities for synergistic combination therapy aimed at achieving “one TCM-multiple targets” for future interventions in diseases related to ferroptosis.

Regarding clinical applications, there is a lack of information about potential biomarkers specifically applicable for diagnosing ferroptosis in clinical settings (Wang X. et al., 2023). Distinct non-invasive biomarkers are crucial not only for identifying ferroptosis in pathological conditions but also for assessing the pharmacodynamics of innovative anti-ferroptosis therapies and monitoring treatment progress. It would be more meaningful to search for ferroptosis biomarkers that can indicate disease severity. Furthermore, there is limited exploration regarding the parameters that dictate the utilization of ferroptosis inducers or inhibitors. These parameters encompass application conditions, initiation time, dosage, administration form, and duration. Current research on the regulatory influence of TCM on ferroptosis has predominantly focused on animal models and specific cell types, with minimal evaluation of clinical safety and efficacy. Comprehensive preclinical and clinical trials are crucial to validate ferroptosis’s involvement in human physiology and provide the groundwork for TCM advancements in treating ferroptosis-related ailments.

#### 3.11.2 Emerging technologies-assisted TCM

##### 3.11.2.1 Artificial intelligence (AI)-Assisted drug screening and design in TCM

AI, originally termed by John McCarthy in 1956 as the ‘science and engineering of developing intelligent machines’, primarily encompasses the fields of computer science, mathematics, and neuroscience. However, it has also found utility in the realm of drug discovery, as indicated by studies (Yang et al., 2019; Zhu Y. et al., 2022; Lin et al., 2022). Given the time-consuming and labor-intensive nature of conventional drug discovery, there is widespread acknowledgment that AI, especially machine learning, has the potential to enhance predictive accuracy and expedite the drug discovery process. Deep learning, a fusion of machine learning and AI, offers a cost-effective platform for drug discovery by enabling rapid, machine-driven decision-making through artificial neural networks. Currently, AI-driven technologies play a pivotal role in various phases of the drug discovery journey, including target identification, drug formulation, screening, synthesis, and anticipating drug characteristics and mechanisms of action (Yang et al., 2019; Gupta et al., 2021). Furthermore, AI proves invaluable in swiftly identifying lead compounds derived from plants and microorganisms (Li G. et al., 2022). Notably, AI has made substantial contributions to cancer research and precision medicine (Bhinder et al., 2021). Within the framework of ferroptosis, Wu et al. employed a blend of bioinformatics and AI to posit that ferroptosis and the TGF-β signaling pathway potentially underpin the protective attributes of celastrol against type 2 diabetes (Wu and Zhang, 2022). In October 2023, Liu et al. demonstrated the potential of deep-learning-assisted phenotypic screening to identify promising lead compounds for alleviating doxorubicin-induced cardiomyopathy by preventing ferroptosis (Liu C. et al., 2023). Their research introduces innovative viewpoints regarding drug discovery in the epoch of AI. As a result, it is plausible to foresee that AI will notably hasten the exploration of innovative TCMs aimed at modulating ferroptosis.

##### 3.11.2.2 Multi-omics

Unlike modern pharmaceuticals, TCM formulations adhere to the TCM theory and the “monarch, minister, assistant, envoy " principle. They constitute comprehensive systems with the capacity for “multiple ingredients, multiple targets, and multiple pathways” (Yi and Chang, 2004; Wang and Zhang, 2017). This enables them to exert synergistic effects in preventing and treating diseases. The complexity of TCM lies in its components, including structurally similar ones, metabolites, and bioactivities. The intricacy of these processes has presented obstacles in the exploration of TCM’s therapeutic mechanisms across diverse diseases. The advent of high-throughput sequencing technology has effectively addressed the requirements of TCM research. Systems biology-driven omics approaches have emerged as viable tools for decoding intricate components, targets, and drug-disease interactions (Subramanian et al., 2020). Multi-omics approaches include various high-throughput analytical methods used in contemporary biological research systems, including genomics, transcriptomics, metagenomics, metabolomics, epigenomics, and proteomics (Olivier et al., 2019; Subramanian et al., 2020). From an omics standpoint, TCM can be regarded as a blend of small molecules. Consequently, a single TCM concoction has the potential to address numerous ailments by leveraging the small molecules and their corresponding gene/protein targets. This shift has significantly advanced the digitization and globalization of TCM (Zhu X et al., 2022).

Network pharmacology analysis stands out as one of the most successful applications in omics investigations (Yang H-Y. et al., 2022; Zhu Y. et al., 2022; Zhu X. et al., 2022; Zhao et al., 2023). TCM formulations usually comprise a variety of components and target multiple biological pathways, with the aim of achieving synergistic effects in treating diseases. This aligns with the fundamental principle of network pharmacology analysis (Zhang et al., 2019; Yang P. et al., 2022), which is dedicated to elucidating the specific compounds within TCM formulations responsible for therapeutic effects and identifying potential targets for drug development (Boezio et al., 2017; Wu et al., 2018; Zhang et al., 2019; Gan et al., 2023). Consequently, network pharmacology analysis has evolved into a powerful approach, leveraging the intricate network of interactions among ingredients, compounds, proteins/genes, and diseases to advance TCM research (Zhang et al., 2019; Yang P. et al., 2022). It offers an effective means to explore the complex interactions between TCM and diseases. To date, several pioneering researchers have investigated the regulatory mechanism of TCM formulations on ferroptosis using omics methodologies. This has marked a transition in TCM research from the traditional “one-target, one-drug” model to a “multi-target, multi-component” framework (Ding et al., 2021; Liu et al., 2021; Zhang Q. et al., 2022; Chen J. et al., 2023; Liu S. et al., 2023; Wang Y. et al., 2023; Wang et al., 2023d; Gu et al., 2023; Qin et al., 2023; Wu et al., 2023; Bai et al., 2024; Shi et al., 2024).

However, applying the multi-omics approach to TCM comes with several limitations (Zhu X. et al., 2022). Network analyses, for instance, are still in their early stages and can sometimes be misleading. These limitations encompass various aspects. First, the vast amount of heterogeneous data, characterized by high dimensionality and complexity, poses a formidable challenge for TCM analysis. Second, a scarcity of data resources hinders a comprehensive comprehension of TCM formulations. This encompasses the identification of herbal components and their bioactive compounds, as well as the elucidation of interactions among small molecules within herbal formulas and human genes and proteins. The complex chemical composition of TCM further complicates both the characterization of their bioactive compounds and the study of their systemic effects in humans. Thirdly, a constrained comprehension of biochemistry has impeded a more profound insight into the pharmacological and toxicological impacts of TCM. Furthermore, despite the extensive utilization of multi-omics in TCM research and clinical contexts, augmenting our understanding of the connections between TCM and diseases could be achieved through the integration of supplementary digital data, encompassing protein structural information, biological visuals, and electronic health records. Finally, while omics approaches have been extensively employed in new drug discovery for TCM, the pharmacodynamic effects of these new drugs should be rigorously evaluated through clinical trials. In future studies, it is imperative that bioinformatics experts with strong clinical backgrounds serve as intermediaries, facilitating the integration of multi-omics methodologies and datasets into established clinical practices to enhance clinical diagnostics. We are confident that these efforts will prove beneficial in advancing the modernization, global integration, and digitization of TCM to address a myriad of complex medical conditions.

##### 3.11.2.3 Nano-TCM

While TCM and its natural components show potential in treating various diseases, their clinical application faces challenges such as limited administration methods, low solubility, instability, brief biological half-life, limited targeting, facile metabolism, and swift elimination (Thanki et al., 2013; Wei et al., 2022). Therefore, the concept of nano-TCM has been introduced as an innovative strategy to enhance the clinical applicability of TCM. Nanotechnology confers substantial benefits in surmounting these impediments (Zhang C. et al., 2020; Shan et al., 2020; Wong et al., 2020; Wang J. et al., 2021; Zheng et al., 2021; Zhang S. et al., 2022). Nanotechnology can enhance the bioavailability and specificity of TCM while concurrently reducing adverse reactions through nano-processing (Huang et al., 2023).

First, nano drug delivery systems (NDDSs) improve the permeability of TCMs, facilitating their transport across physiological barriers such as the skin, mucosa, and blood-brain barrier (Arvanitis et al., 2020; Hakim et al., 2020; Paiva-Santos et al., 2021). This improved transport occurs through transcellular, paracellular, and carrier-mediated pathways (Khan et al., 2015). Secondly, NDDSs facilitate the prolonged or regulated release of TCMs (Moradi Kashkooli et al., 2020). Prolonged release extends the duration of therapeutic effects, ensuring consistent release rates, lessening the need for frequent dosing, and mitigating side effects. Controlled release, accomplished via external means (such as ultrasound, near-infrared light, or microwaves) or internal factors (such as the microenvironment conditions at the lesion site, including pH and specific enzymes), reduces toxicity in healthy tissues while optimizing therapeutic efficacy (Hossen et al., 2019; Jiang and Zhang, 2023). Thirdly, NDDSs upgrade the bioavailability of hydrophobic TCM ingredients. These NDDSs greatly augment the bioavailability of TCM components by enhancing their aqueous solubility and biocompatibility (Bilia et al., 2019). Lastly, NDDSs enable the co-delivery of different TCM ingredients, harnessing synergistic effects and allowing for lower and safer doses. Various NDDSs, including polymer-based, mesoporous silica, and lipid-based carriers, have been utilized for this purpose (Gurunathan et al., 2018; Cano et al., 2020; Paris and Vallet-Regí, 2020).

In the field of “ferroptosis-TCM”, a biomimetic NDDS was designed by a team of researchers from Nanjing Medical University. This system involved the creation of nanoparticles (NPs) loaded with resveratrol and coated with erythrocyte membrane. The objective was to facilitate the evasion of macrophage phagocytosis, leading to an extended circulation period and ultimately enhancing ferroptosis-induced anticancer effects (Zhang Z. et al., 2022). In the same year, researchers from Shanghai University of Traditional Chinese Medicine developed camptothecin (CPT) NPs (Su et al., 2022). These NPs were designed as a NDDS consisting of CPT-loaded polydopamine, coated with manganese dioxide. This innovative approach enabled both diagnosis and therapy, utilizing magnetic resonance imaging and chemo-photothermal therapy, respectively.

However, nano-TCM is still in its infancy. Nano-TCM is currently characterized by a lack of guidance from TCM theory and inadequate basic research. Nanotechnology has altered or controlled certain physical and chemical reactions among TCM ingredients, introducing uncertainty regarding the active components of TCM. This poses a challenge to TCM theory. Therefore, it is crucial to gain a deeper understanding of metabolic pathways to ensure the clinical safety of nanocarriers and guide their systematic design (Wei et al., 2022). Secondly, although nanotechnology’s application in TCM research has expanded the possibilities for TCM modernization, there remains a pressing need to bolster fundamental and exploratory research. Urgent attention should be given to addressing the deficiencies in nanotechnology preparation techniques, the insufficiencies in evaluating pharmacological efficacy, and the discrepancies between the prerequisites for encapsulating pharmacodynamic elements of TCM and nanoparticles NPs. Furthermore, the clinical efficacy of nanodrugs raises thought-provoking concerns (Cabral et al., 2023). Over the past 3 decades, nanodrugs have proliferated, with increasingly complex designs. However, only ten nanodrugs have received approval from the US Food and Drug Administration, and only 14% of these medications have demonstrated enhanced clinical efficacy (He et al., 2019). Finally, the safety of NPs is a critical issue that requires thorough investigation (Zhang et al., 2021; Chen Y. et al., 2023; Lu et al., 2023). Although the majority of safety assessments for nanocarriers are conducted in diverse cell lines and animal models, human biological responses can exhibit variability, which constrains the applicability of safety evaluations solely reliant on animal research.

To advance TCM, it is essential to promote interdisciplinary cooperation and facilitate the fusion of TCM with modern scientific and technological methods. While adhering to the core principles of TCM theory, it is crucial to foster creativity and advancements to revitalize its practical applications. NDDSs present a chance to investigate the utilization of TCM, and the amalgamation of TCM with nanotechnology can provide inventive approaches and viewpoints to propel the modernization of TCM, consequently bolstering its sustained growth (Sun R. et al., 2022; Wei et al., 2022; Huang et al., 2023).

#### 3.11.3 Future development prospects

TCM has shown significant potential in the regulation and treatment of human diseases; however, further exploration in this research area is needed. Currently, “ferroptosis-TCM” research faces three major challenges. Firstly, studies on TCM are mostly limited to the treatment of active ingredients or herbal monomers, with few investigations into TCM compound prescriptions, proprietary Chinese medicines, and external treatment methods (Xie et al., 2023; Chen et al., 2024). Secondly, there are four broad mechanisms have been identified for inducing ferroptosis (Dixon et al., 2012): inhibition of the system Xc^−^ (Sun et al., 2023), inhibition/degradation/inactivation of GPx4 (Wang X. et al., 2023), depletion of reduced coenzyme Q_10_, and (Friedmann Angeli et al., 2019) induction of lipid peroxidation through peroxides, iron, or polyunsaturated fatty acid overload. A great deal of TCMs induce tumor ferroptosis via different mechanisms (Wang et al., 2023b). However, these investigations often focus on a single regulatory pathway mentioned above, neglecting the cross-dialogue within the overall regulatory networks of ferroptosis. A similar issue is observed in the domain of anti-ferroptotic TCMs. Thirdly, different diseases have distinct therapeutic targets. Promoting ferroptosis in tumor cells aids in inhibiting and killing tumors, but normal cells like cardiomyocytes, pancreatic β-cells, and neurons are also sensitive to ferroptosis. Addressing the selective regulation of ferroptosis is a major issue that requires extensive future research.

In the future, efforts should integrate clinical TCM practice with basic research. Exploring the molecular mechanisms of TCM in regulating ferroptosis from multiple channels and perspectives is essential. Additionally, there is a need to increase the study of ferroptosis-related mechanisms in TCM compound prescriptions, proprietary Chinese medicines, and external treatments with remarkable clinical effects. This exploration aims to understand the specific mechanisms of preventing and treating human diseases by targeting ferroptosis, contributing to the development of new theoretical foundations and potential therapeutic strategies for clinical treatment.

### 3.12 Strengths and limitations

In contrast to previous investigations primarily limited to systematic or narrative reviews, the combination of scientometric and visualized analysis provides readers with a clearer representation of research focal points and trends across various dimensions (Nguyen et al., 2021; Zhang J. et al., 2022). In its role as the inaugural bibliometric analysis endeavoring to chart the knowledge landscape of “ferroptosis-TCM” during the last 11 years. This research provides a relatively exhaustive and unbiased point of reference, notwithstanding inherent limitations.

This study has quite a few limitations: 1) The use of CiteSpace, which is limited to WoSCC publications, may introduce selection bias due to the software’s inherent limitations (Ling et al., 2023). 2) Citation bursts, influenced by factors like publication date and journal quality, may not accurately reflect a paper’s influence. 3) The inability to comprehensively review and analyze all papers and their subfields necessitated equal attention to both high and low-quality publications, potentially impacting the study’s credibility. 4) Scientometric methods, reliant on natural language processing, may manifest biases, as evidenced in previous studies (Zhang et al., 2018; Yan et al., 2021; Zhang J. et al., 2022; Cao et al., 2023b; Cao et al., 2024). 5) Limiting the research to English-language documents may introduce publication bias. 6) Incomplete retrieval of recent literature and keywords may impact the results due to gaps in literature collection. 7) VOSviewer automatically extracts author names, which may not always be extracted accurately. Some authors may use different name spellings or multiple names, potentially leading to inaccuracies in research results for these authors (Peng et al., 2022).

## 4 Conclusion

The present study conducts a trend analysis of “ferroptosis-TCM” within the biomedical field, spanning the years 2012–2023 and utilizing scientometric methodologies. The analysis specifically focuses on international collaboration, publication trends, and research hotspots. These findings serve to empower the scientific community by identifying emerging ideas and frontiers that will shape the future of ferroptosis research for TCM. For researchers, keeping abreast of these trends and utilizing existing knowledge to propel progress in this field is paramount. However, “ferroptosis-TCM” faces numerous pressing issues, including, but not limited to (Dixon et al., 2012): the elusive primary factor responsible for triggering ferroptosis following lipid peroxidation (Sun et al., 2023); the inadequate exploration of TCM, encompassing both ferroptosis inducers and inhibitors, with regard to conditions of application, initiation time point, dose, administration form, and duration (Wang X. et al., 2023); the current understanding of “ferroptosis-TCM” relies primarily on data from animal or cellular studies, with limited evaluation of its clinical safety and efficacy; and (Friedmann Angeli et al., 2019) crosstalk exists between ferroptosis and other types of PCD. The targeting of ferroptosis heralds a new era for TCM, with the potential to revolutionize healthcare despite the existing challenges. To overcome these challenges and fully leverage the potential of “ferroptosis-TCM” within this field, increased efforts and enhanced collaboration are imperative across the fields of pharmacology, biology, basic science, and clinical medicine.

## Data Availability

The raw data supporting the conclusion of this article will be made available by the authors, without undue reservation.
