# Supplementary material for: Targeting ferroptosis unveils a new era for traditional Chinese medicine: a scientific metrology study
Source: Front Pharmacol. 2024 Feb 14;15:1366852. doi: 10.3389/fphar.2024.1366852 (PMC10921231; doi:10.3389/fphar.2024.1366852)
Supplement: Supplementary file 1 [file Table1.DOCX]

Supplementary Material

**Targeting Ferroptosis Unveils a New Era for Traditional Chinese Medicine: A Scientific Metrology Study**

**Siyang Cao^1, 2, 3,^** **^†^, Yihao Wei^1, 2, 3, †^, Yaohang Yue^1, 2, 3, †^, Yingqi Chen^1, 2, 3^, Shuai Liao^1, 2, 3^, Aikang Li^1, 2, 3^, Peng Liu^1, 2, 3, *^, Ao Xiong^1, 2, 3, *^, Hui Zeng^1, 2, 4, *^**

1 National & Local Joint Engineering Research Centre of Orthopaedic Biomaterials, Peking University Shenzhen Hospital, Shenzhen, Guangdong, People's Republic of China.

2 Shenzhen Key Laboratory of Orthopaedic Diseases and Biomaterials Research, Peking University Shenzhen Hospital, Shenzhen, Guangdong, People's Republic of China.

3 Department of Bone & Joint Surgery, Peking University Shenzhen Hospital, Shenzhen, Guangdong, People's Republic of China.

4 Shenzhen Second People's Hospital, The First Affiliated Hospital of Shenzhen University, Shenzhen, Guangdong, People's Republic of China.

***Correspondence authors:**

Peng Liu (liupeng_polymer@126.com), Ao Xiong (xiongao@189.cn), and Hui Zeng (zenghui@pkuszh.com).

**†**Siyang Cao, Yihao Wei, and Yaohang Yue contributed equally to this work and share first authorship.

*Peng Liu, Ao Xiong, and Hui Zeng contributed equally to this work and share last authorship.

# Search strategy

| **Search** | **Query** |
| --- | --- |
| #1 | TS=(ferroptosis OR “iron death” OR “iron overload”) |
| #2 | TS=("Medicine, Chinese Traditional" or "Traditional Chinese Medicine" or "Chung I Hsueh" or "Hsueh, Chung I" or "Traditional Medicine, Chinese" or "Zhong Yi Xue" or "Chinese Traditional Medicine" or "Chinese Medicine, Traditional" or "Traditional Tongue Diagnosis" or "Tongue Diagnoses, Traditional" or "Tongue Diagnosis, Traditional" or "Traditional Tongue Diagnoses" or "Traditional Tongue Assessment" or "Tongue Assessment, Traditional" or "Traditional Tongue Assessments" or "Chinese herbal medicine" or "Chinese herbal medicines" or "Chinese medicine monomer" or "Chinese medicine monomers" or "Chinese herbal compound" or "Chinese herbal compounds" or "Plant Extracts" or "Extracts, Plant" or "Plant Extract" or "Extract, Plant" or "Herbal Medicines" or "Medicines, Herbal" or "PHYTOMEDICINE" or "phytopharmaceuticals" or "phytopharmaceutical" or "Chinese herbs" or "Chinese herb" or "Phenolic compounds" or "Phenolic compound" or "Quinones" or "Flavonoids" or "2-Phenyl-Chromenes" or "2 Phenyl Chromenes" or "2-Phenyl-Benzopyran" or "2 Phenyl Benzopyran" or "2-Phenyl-Benzopyrans" or "2 Phenyl Benzopyrans" or "2-Phenyl-Chromene" or "2 Phenyl Chromene" or "Flavonoid" or "Bioflavonoids" or "Bioflavonoid" or "Polyphenols" or "Polyphenol" or "Provinols" or "Alkaloids" or "Alkaloid" or "Plant Alkaloids" or "Alkaloids, Plant" or "Plant Alkaloid" or "Alkaloid, Plant" or "Saponins" or "Saponin" or "Terpenes" or "Terpenoids" or "Terpene" or "Terpenoid" or "Isoprenoids" or "Isoprenoid" or "Polysaccharides" or "Polysaccharide" or "Glycans" or "Glycan" or "Chrysophanol") |
| #3 | #1 AND #2 |
